# Computational Assessment of Thermal and Solute Mechanisms in Carreau–Yasuda Hybrid Nanoparticles Involving Soret and Dufour Effects over Porous Surface

**DOI:** 10.3390/mi12111302

**Published:** 2021-10-23

**Authors:** Enran Hou, Fuzhang Wang, Essam Roshdy El-Zahar, Umar Nazir, Muhammad Sohail

**Affiliations:** 1 College of Mathematics, Huaibei Normal University, Huaibei 235000, China; 2 Nanchang Institute of Technology, Nanchang 330044, China; wangfuzhang1984@163.com; 3 School of Mathematical and Statistics, Xuzhou University of Technology, Xuzhou 221018, China; 4 Department of Mathematics, College of Science and Humanities in Al-Kharj, Prince Sattam bin Abdulaziz University, P.O. Box 83, Al-Kharj 11942, Saudi Arabia; er.elzahar@psau.edu.sa; 5 Department of Basic Engineering Science, Faculty of Engineering, Menoufia University, Shebin El-Kom 32511, Egypt; 6 Department of Applied Mathematics and Statistics, Institute of Space Technology, P.O. Box 2750, Islamabad 44000, Pakistan; nazir_u2563@yahoo.com

**Keywords:** viscous dissipation, chemical reaction, finite element procedure, hybrid nanoparticles, heat and mass transfer rates, joule heating

## Abstract

Engineers, scientists and mathematicians are greatly concerned about the thermal stability/instability of any physical system. Current contemplation discusses the role of the Soret and Dufour effects in hydro-magnetized Carreau–Yasuda liquid passed over a permeable stretched surface. Several important effects were considered while modelling the thermal transport, including Joule heating, viscous dissipation, and heat generation/absorption. Mass transportation is presented in the presence of a chemical reaction. Different nanoparticle types were mixed in the Carreau–Yasuda liquid in order to study thermal performance. Initially, governing laws were modelled in the form of PDEs. Suitable transformation was engaged for conversion into ODEs and then the resulting ODEs were handled via FEM (Finite Element Method). Grid independent analysis was performed to determine the effectiveness of the chosen methodology. Several important physical effects were explored by augmenting the values of the influential parameters. Heat and mass transfer rates were computed against different parameters and discussed in detail.

## 1. Introduction

The mechanism of transport phenomenon in different materials has received reasonable attention recently due to its wider applications in industry and different medical processes. Several important materials exist for the support of these mechanisms. Due to their different characteristics, these materials cannot be explained through one constitutive relation. Carreau–Yasuda is one such important material which has the following constitute relation.

For Υ=0 or n=1, the Newtonian model is recovered. This model predicts the relation of shear stress with frequency. Several important contributions have been made by considering this material. For example, Zare et al. [1] discussed this model by experimentally considering the complex viscosity relationship. In their investigation, they found an excellent settlement of frequency data. They considered the involvement of carbon nanotubes in the mixture of Carreau–Yasuda material. Kayani et el. [2] reported on the behavior of wall properties on the peristaltic flow of the Carreau–Yasuda model in a sinusoidal channel by considering the Hall effect. Governing laws for the transport of species, heat and momentum were modeled under the low-Reynolds-number assumption along with the long wavelength approach. After implementing the scaling group transformation, the transformed problem was approximated numerically via the ND-Solve tool in the MATHEMATICA 15.0 symbolic package. The authors conducted a parametric analysis and their findings were shown in several graphs. They noticed a decline in the thermal field for the Biot number and recorded an enhancement in the mounting values of the Brinkman number. The unsteady rheology of the Carreau–Yasuda material in a circular tube was examined by Rana and Murthy [3]. In their investigation, they reported the wall absorption effect. They retrieved different flow behavior cases by considering different values of material parameters. Sochi [4] presented the modelling of Cross and Carreau liquid through a circular pipe. Analytical and numerical schemes were jointly implemented for the solution of flow equations and an excellent settlement was monitored. The rheology of the Carreau–Yasuda model in a cavity at high-Reynolds-number was examined by Shamekhi and Aliabadi [5] via the mesh-free algorithm. The phenomenon of blood flow via the Carreau–Yasuda model was reported by Jahangiri [6] through the FEM package.


(1) $$\eta_{CY}\left(\dot{\gamma }\right)=\mu_{\infty }+\left(\mu_{0}-\mu_{\infty }\right){\left[1+{\left(\Upsilon \dot{\gamma }\right)}^{d}\right]}^{\frac{n-1}{d}}.$$

The involvement of nanoparticles enhanced the thermal performance and heat transportation rate. Several models of the nanomaterials are available and frequently used to study the thermal performance of different materials. Several researchers have paid attention to these materials due to their wider applications and usage. For instance, Gorla and Gireesha [7] developed the modeling of steady viscoelastic material with convective heat transport. The Buongiorno model is utilized to capture the characteristics of Brownian diffusion and thermophoresis. Modelling of heat transport is carried out by considering thermal radiation and heat generation. They solved boundary layer equations via a shooting procedure in the MATLAB symbolic package. The impact of several pertinent parameters were displayed through graphs, and tabular results were prepared to demonstrate the effectiveness and applicability of the shooting scheme for a large set of nonlinear data arising in the mechanical engineering problem. Muhammad et al. [8] modelled the squeezed flow of a viscous nanofluid with an updated mass and heat fluxes between parallel plates and handled the resulting expressions analytically via the OHAM in MATHEMATICA 15.0 computational tool. They noticed the enhancement in fluid velocity for the larger squeezing parameter. Rashid et al. [9] presented the exact solution of a water-based mixture of aligned nanoparticle materials under the radiation effect. They recorded the depreciation in heat transfer rate against the radiation and slip parameter. The double stratification phenomenon in the buoyancy-driven flow of a micro-polar viscous nanofluid was examined by Ramzan et al. [10]. They analyzed the depreciation in velocity for the buoyancy parameter and an enhancement was recorded against the micro-polar parameter. Hezma et al. [11] studied the behavior of SWCNTs in order to investigate the mechanical properties of polyvinyl chloride. Upadhay and Raju [12] examined the inclusion of dust particles in Eyring–Powell material over a stretched sheet. They studied the thermal and mass transport in dusty Eyring–Powell nanofluid by engaging the revised definitions of mass and heat fluxes. They found the numerical solutions for nonlinear modeled equations via the shooting scheme. Several important pieces of research on the transport phenomenon are reported in [13,14,15,16,17] and the references therein. Nazir et al. [18] studied comparison analysis among hybrid nanoparticles and nanoparticles in hyperbolic tangent liquid past a stretching surface. They adopted a finite element approach to conduct numerical results. Chu et al. [19] modeled correlations between nanoparticles and hybrid nanoparticles, considering activation energy and chemical reaction. They noticed thermal aspects past a parabolic surface using a finite element scheme. Cui et al. [20] simulated effects related to radius and roughness of inserting nanoparticles. Awais et al. [21] applied the KKL model in the transfer of energy using nanoparticles. Nazir et al. [22] discussed the numerical results of the Carreau–Yasuda liquid in heat/energy considering the hybrid nanoparticles and nanomaterials, numerically solved by the finite element approach.

**Figure 1:**
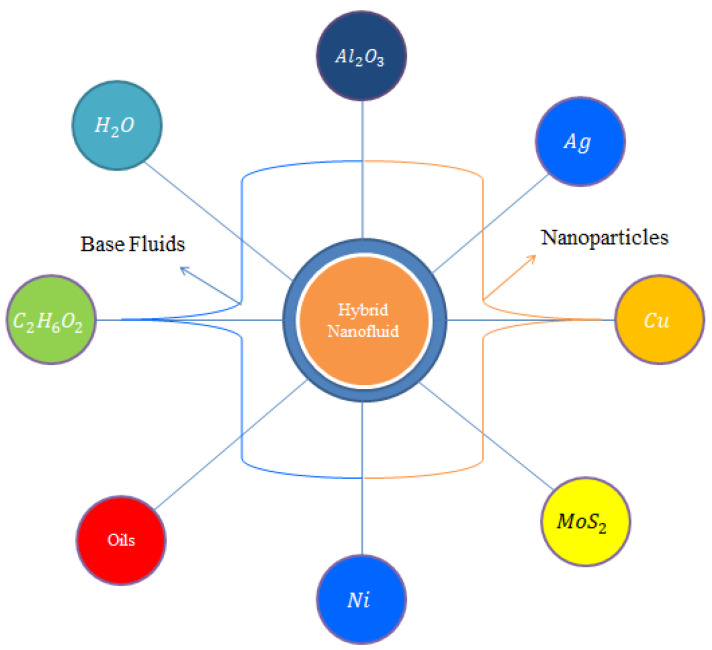
The sketching behavior of hybrid nanoparticles.

In the above cited literature, no study deals with the combined behavior of the following: mass, heat transport in hydro-magnetized Carreau–Yasuda material using Joule heating, viscous dissipation, heat generation, chemical reaction and the Soret and Dufour influences in the Darcy–Forchheimer porous stretching sheet. This report fills the gap in this discussion and should be used as a foundation for researchers to work further on this model. The inclusion of nanoparticles in the Carreau–Yasuda material is attractive to researchers. Organization of this research is divided in the following way: the literature survey is reported in Section 1, modelling with important physical assumptions are covered in Section 2, Section 3 covers a detailed description of the finite element procedure with a grid independent survey, a detailed description of the solution and the influence of several emerging parameters are explained in Section 4 and important findings of the reported study are listed in Section 5. Figure 1 reveals the division of the base fluids, hybrid nanoparticles and nanoparticles. In this Figure, $Ag,\;Cu,\;Al_{2}O_{3},\;Ni$ and $MoS_{2}$ are known as nanoparticles whereas $H_{2}O,$ ethylene glycol and oils are called base fluids. In this current analysis, ethylene glycol is considered as a base fluid. Mixtures of $MoS_{2}$ and $SiO_{2}$ are hybrid nanoparticles.

## 2. Development of the Flow Model

**Figure 2:**
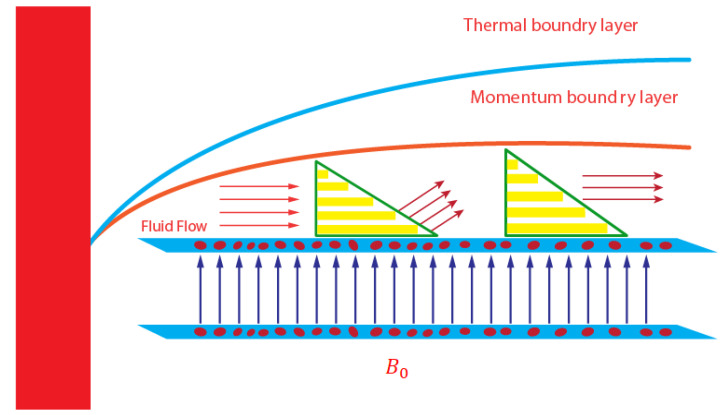
Geometry of transport phenomenon in Carreau–Yasuda fluid.

**Figure 3:**
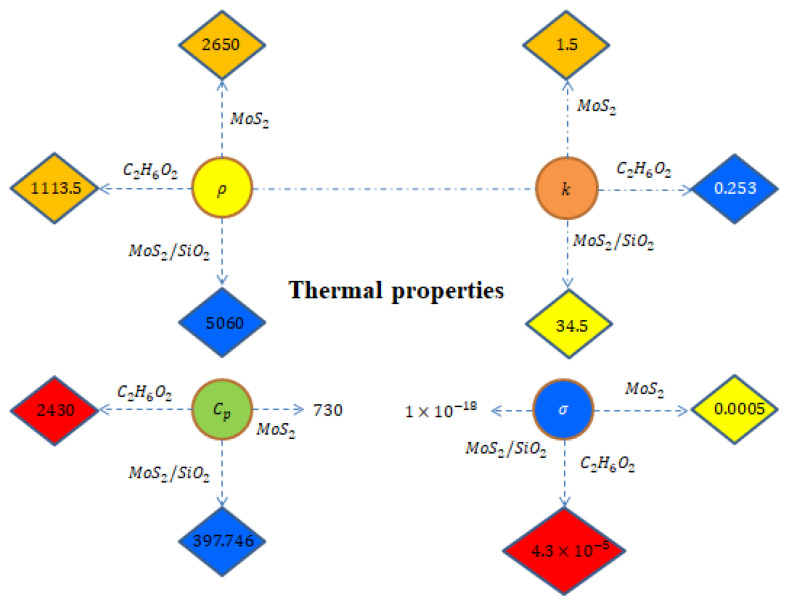
Thermal properties of nanoparticles and hybrid nanoparticles in base liquid.

An enhancement in the thermal and solute performance of Carreau–Yasuda rheology, inserting the impact of nanoparticles and hybrid nanoparticles, is considered as shown in Figure 1. The flow runs towards the stretching surface under the action of a constant magnetic field. Heat takes place due to Joule heating and viscous dissipation. The Soret and Dufour influences are captured with heat generation and chemical reaction. Forchheimer’s porous theory is imposed in the transport phenomenon. The geometrical flow diagram is considered in Figure 2 and the thermal properties of the nanoparticles are shown in Figure 3.

The non-linear PDEs are developed according to the physical happenings and the boundary layer approximations.

(2) $$\frac{\partial \tilde{u}}{\partial x}+\frac{\partial \tilde{v}}{\partial y}=0,$$

(3) $$\tilde{u}\frac{\partial \tilde{u}}{\partial x}+\tilde{v}\;\frac{\partial \tilde{u}}{\partial y}+\frac{\nu_{hnf}}{k^{a}}F_{s}\tilde{u}+\frac{F_{s}}{{\left(k^{a}\right)}^{1/2}}{\left(\tilde{u}\right)}^{2}=\nu_{hnf}\left[\frac{\partial^{2}\tilde{u}}{\partial y^{2}}+{\left(\Lambda \right)}^{d}\left(\frac{m-1}{d}\right)\left(d+1\right)\frac{\partial^{2}\tilde{u}}{\partial y^{2}}{\left(\frac{\partial \tilde{u}}{\partial y}\right)}^{d}\right]$$

(4) $$-\frac{B_{0}^{2}\sigma_{hnf}}{\rho_{hnf}}\tilde{u}\sin^{2}\alpha ,$$

(5) $$\tilde{u}\frac{\partial \tilde{T}}{\partial x}+\tilde{v}\;\frac{\partial \tilde{T}}{\partial y}-\frac{Q}{{\left(\rho C_{p}\right)}_{hnf}}\left(\tilde{T}-T_{\infty }\right)-\frac{D_{hnf}k_{T}}{{\left(C_{p}\right)}_{f}C_{s}}\frac{\partial^{2}\tilde{C}}{\partial y^{2}}=\frac{k_{hnf}}{{\left(\rho C_{p}\right)}_{hnf}}\frac{\partial^{2}\tilde{T}}{\partial y^{2}}+\frac{B_{0}^{2}\sigma_{hnf}}{{\left(\rho C_{p}\right)}_{hnf}}\sin^{2}\alpha {\left(\tilde{u}\right)}^{2}$$

(6) $$+\frac{\mu_{hnf}}{{\left(\rho C_{p}\right)}_{hnf}}\left[{\left(\Lambda \right)}^{d}\left(\frac{m-1}{d}\right){\left(\frac{\partial \tilde{u}}{\partial y}\right)}^{d}\right]{\left(\frac{\partial \tilde{u}}{\partial y}\right)}^{2},$$

(7) $$\tilde{u}\frac{\partial \tilde{C}}{\partial x}+\tilde{v}\;\frac{\partial \tilde{C}}{\partial y}=D_{hnf}\frac{\partial^{2}\tilde{C}}{\partial y^{2}}-k_{0}\left(\tilde{C}-C_{\infty }\right)+\frac{D_{hnf}k_{T}}{T_{m}}\frac{\partial^{2}\tilde{T}}{\partial y^{2}},$$

where $\left(\tilde{u},\;\tilde{v}\right)$ is velocity componets, space coordinates are (x, y), kinamtic viscosity is ν, $F_{s}$ is inertia cofficient (porous medium), permeability (porou medium) is $k^{a},$ power law index number is m, time constant is Λ, magnetic induction is $B_{0}$, electrical conductivity is σ, temprature is $\tilde{T}$, $\tilde{C}$ is the concentration, heat source is Q, $T_{\infty }$ is ambient temprature, fluid density is ρ, $\tilde{C}$ is concentarion, mass diffusion is D, $T_{m}$ is fluid mean temprature, $k_{T}$ is thermal diffusion, $C_{s}$ is concentration susceptibility and $k_{0}$ is chemical reaction number.

The no-slip theory provides the required boundary conditions of the current model:

$$\tilde{u}=ax,\;\tilde{v}=0,\;\tilde{C}=C_{w},\;\tilde{T}=T_{w}:\;y=0,$$

(8) $$\tilde{u}\to 0,\;\tilde{C}\to C_{\infty },\;\tilde{T}\to T_{\infty }:y\to \infty .$$

Change in variables is constructed as:

(9) $$\tilde{u}=axF^{\prime },\;\tilde{v}=-{\left(a\nu_{f}\right)}^{\frac{1}{2}}F,\;\xi ={\left(\frac{a}{\nu_{f}}\right)}^{\frac{1}{2}}y,$$

(10) $$\theta \left(T_{w}-T_{\infty }\right)=\tilde{T}-T_{\infty },\;\left(C_{w}-C_{\infty }\right)\phi =\tilde{C}-C_{\infty }.$$

Transformations are used in Equations (1)–(5) and the system of non-linear PDEs are converted to following ODEs

(11) $$\begin{array}{c} F_{\xi \xi \xi }+{\left(We\right)}^{d}\frac{\left(m-1\right)\left(d+1\right)}{d}F_{\xi \xi \xi }{\left(F_{\xi \xi }\right)}^{d}+A_{1}\left(FF_{\xi \xi }-F_{\xi }^{2}\right)-\epsilon F_{\xi }-A_{1}F_{r}{\left(F_{\xi }\right)}^{2}\\ -A_{2}M^{2}\sin^{2}\alpha F_{\xi }=0,\\ F\left(0\right)=0,\;F_{\xi }\left(0\right)=1,F_{\xi }\left(\infty \right)=1, \end{array}\},$$

(12) $$\begin{array}{c} \theta_{\xi \xi }+A_{3}PrF\theta_{\xi }+A_{4}H_{s}\theta +A_{4}M^{2}Ec\sin^{2}\alpha {\left(F_{\xi }\right)}^{2}+A_{5}PrEC\left[1+{\left(We\right)}^{d}\frac{\left(m-1\right)}{d}{\left(F_{\xi \xi }\right)}^{d}\right]{\left(F_{\xi \xi }\right)}^{2}\\ +A_{4}A_{6}PrD_{f}\phi_{\xi \xi }=0,\\ \theta \left(0\right)=1,\;\theta \left(\infty \right)=0, \end{array}\},$$

(13) $$\begin{array}{c} \varphi_{\xi \xi }+\frac{Sc}{{\left(1-\phi_{2}\right)}^{2.5}{\left(1-\phi_{1}\right)}^{2.5}}F\varphi_{\xi }-\frac{K_{c}Sc}{{\left(1-\phi_{2}\right)}^{2.5}{\left(1-\phi_{1}\right)}^{2.5}}\varphi +ScSr\theta_{\xi \xi }=0,\\ \varphi \left(0\right)=1,\;\varphi \left(\infty \right)=0, \end{array}\}.$$

Here, $A_{1},\;A_{2},\;A_{3},\;A_{4},\;A_{5}$ and $H_{1}$ are involved parameters (representing the correlation of nanoparticles and hybrid nanostructures) in the above equations which are defined as

(14) $$A_{1}={\left(1-\phi_{1}\right)}^{2.5}{\left(1-\phi_{2}\right)}^{2.5}\left[\left(1-\phi_{2}\right)\left\{\left(1-\phi_{1}\right)+\phi_{1}\frac{\rho_{s_{1}}}{\rho_{f}}\right\}\right]+\phi_{2}\frac{\rho_{s_{2}}}{\rho_{f}},$$

(15) $$A_{2}={\left(1-\phi_{1}\right)}^{2.5}{\left(1-\phi_{2}\right)}^{2.5},A_{4}=\frac{k_{f}}{k_{hnf}},A_{5}=\frac{k_{f}}{{\left(1-\phi_{1}\right)}^{2.5}{\left(1-\phi_{2}\right)}^{2.5}k_{hnf}},$$

(16) $$A_{3}=A_{4}\left[\left(1-\phi_{2}\right)\left\{\left(1-\phi_{1}\right)+\phi_{1}\frac{{\left(\rho C_{p}\right)}_{s_{1}}}{{\left(\rho C_{p}\right)}_{f}}\right\}\right]+\phi_{1}\frac{{\left(\rho C_{p}\right)}_{s_{2}}}{{\left(\rho C_{p}\right)}_{f}},$$

(17) $$A_{6}={\left(1-\phi_{1}\right)}^{2.5}{\left(1-\phi_{2}\right)}^{2.5}\left[\left(1-\phi_{2}\right)\left\{\left(1-\phi_{1}\right)+\phi_{1}\frac{{\left(\rho C_{p}\right)}_{s_{1}}}{{\left(\rho C_{p}\right)}_{f}}\right\}\right]+\phi_{1}\frac{{\left(\rho C_{p}\right)}_{s_{2}}}{{\left(\rho C_{p}\right)}_{f}},$$

(18) $$\frac{k_{hnf}}{k_{bf}}=\left\{\frac{k_{s_{2}}+\left(n-1\right)k_{bf}-\left(n-1\right)\phi_{2}\left(k_{bf}-k_{s_{2}}\right)}{k_{s_{2}}+\left(n-1\right)k_{bf}-\phi_{2}\left(k_{bf}-k_{s_{2}}\right)}\right\}.$$

Figure 3 demonstrates the thermal properties of density, electrical conductivity, thermal conductivity and specific heat capacitance for ethylene glycol, $MoS_{2}/SiO_{2}$ and $MoS_{2}$. Density of $C_{2}H_{6}O_{2}$ is 1113.5, density of $MoS_{2}$ is 2650, density of $MoS_{2}/SiO_{2}$ is 5060, thermal conductivity of $C_{2}H_{6}O_{2}$ is 0.253, thermal conductivity of $MoS_{2}$ is 1.5, thermal conductivity of $MoS_{2}/SiO_{2}$ is 34.5, electrical conductivity of $MoS_{2}$ is 0.0005, electrical conductivity of $C_{2}H_{6}O_{2}$ is $4.3\times 10^{-5}$, electrical conductivity of $MoS_{2}/SiO_{2}$ is $1\times 10^{-18}$, $C_{p}$ of $C_{2}H_{6}O_{2}$ is 2430, $C_{p}$ of $MoS_{2}$ is 730 and $C_{p}$ of $MoS_{2}/SiO_{2}$ is 397.746, respectively. Here, the Weissenberg number is $We=\left(\frac{\Lambda xa^{3/2}}{{\left(\nu_{f}\right)}^{1/2}}\right)$, the magnetic number is $M^{2}\left(=\frac{B_{0}^{2}\sigma_{f}}{a\rho_{f}}\right)$, the porosity number is $\epsilon \left(=\frac{F_{s}\nu_{f}}{a}\right)$, the Forchheimer number is $F_{r}=\left(\frac{xF_{s}}{\sqrt{k^{*}}}\right)$, the Prandtl number is $Pr\left(=\frac{\mu_{f}{\left(c_{p}\right)}_{f}}{k_{f}}\right)$, the Eckert number is $Ec\left(=\frac{{\left(U_{w}\right)}^{2}}{\left(T_{w}-T_{\infty }\right){\left(c_{p}\right)}_{f}}\right)$, the heat generation number is $H_{s}\left(=\frac{Q}{a\rho_{f}{\left(c_{p}\right)}_{f}}\right)$, the Schmidt number is $Sc\left(=\frac{\nu_{f}}{D_{f}}\right)$, the chemical reaction number is $K_{c}\left(\frac{k_{c}}{a}\right)$, the Dufour number is $D_{f}\left(=\frac{\left(C_{w}-C_{\infty }\right)D_{f}k_{t}}{C_{a}\nu_{f}{\left(c_{p}\right)}_{f}}\right)$ and the Soret number is $Sr\left(=\frac{D_{f}\left(T_{w}-T_{\infty }\right)k_{t}}{\nu_{f}\left(C_{w}-C_{\infty }\right)T_{m}}\right).$ The surface force in attendance of the Carreau–Yasuda liquid at the wall of the melting surface is

(19) $${\left(Re\right)}^{\frac{1}{2}}C_{f}=\frac{-1}{{\left(1-\phi_{1}\right)}^{2.5}{\left(1-\phi_{2}\right)}^{2.5}}\left[1+\frac{m-1}{d}{\left(WeF_{\xi \xi }\left(0\right)\right)}^{2}\right]F_{\xi \xi }\left(0\right),$$

The temperature gradient because of the nano and hybrid nanoparticles is

(20) $$Nu=\frac{xQ_{w}}{k_{f}\left(T-T_{\infty }\right)},\;Q_{w}=-k_{hnf}\frac{\partial T}{\partial y},$$

(21) $${\left(Re\right)}^{-1/2}Nu=\frac{-k_{hnf}}{k_{f}}\theta_{\xi }\left(0\right).$$

Concentration gradient at the surface of the melting sheet is

(22) $$Sh=\frac{xl_{m}}{\left(\tilde{C_{w}}-\tilde{C_{\infty }}\right)D_{hnf}},\;l_{m}=-D_{hnf}\frac{\partial \tilde{C}}{\partial y}{|}_{y=0,}\;{\left(Re\right)}^{-\frac{1}{2}}Sh=-\frac{\varphi^{\prime }\left(0\right)}{{\left(1-\phi_{1}\right)}^{2.5}{\left(1-\phi_{2}\right)}^{2.5}},$$

The local Reynolds number is $Re=\frac{ax^{2}}{\nu_{f}}.$

## 3. Numerical Approach and Convergence Analysis

**Figure 4:**
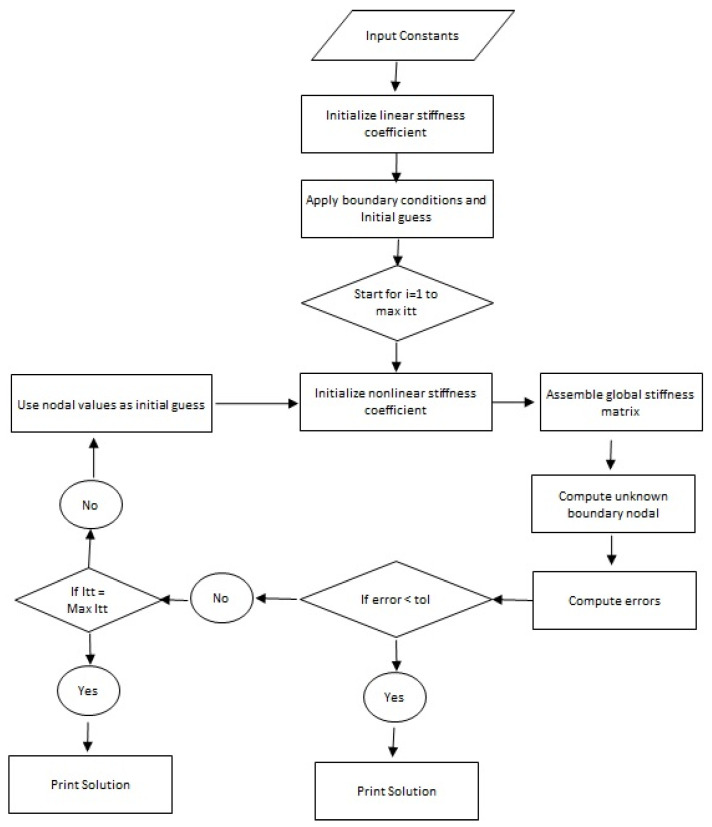
Working scheme of finite element method.

The finite element method is an effective method in the view of accuracy and the convergence of a problem compared with other numerical approaches. There are many advantages to FEM but some are discussed here:

➢ FEM has the ability to handle various complex geometries;
➢ This numerical method is thought to be most significant in solving physical problems with wide ranges;
➢ FEM requires less investment in the view of time and resources;
➢ A main advantage of FEM is its handling of various types of boundary conditions and
➢ It has a good ability with regards to the discretization (of derivative) problems into small elements;
➢ The Working scheme of finite element method has been shown with the help of Figure 4.

The numerical approach called finite element scheme is used to simulate the numerical results of highly non-linear PDEs and numerous applications of FEM are found in CFD (computational fluid dynamics) problems. The FEM approach is explained in the following steps:

**Step I:** The division of a problem domain into a finite number of elements and residuals. The weak form is captured from the strong form due to residuals. The approximation result is simulated using shape functions and the approximation simulations of the variables are:

(23) $$H=\overset{2}{\underset{l=1}{\sum }}\left(H_{l}\omega_{j}\right),\;F=\overset{2}{\underset{l=1}{\sum }}\left(F_{l}\omega_{j}\right),\;\theta =\overset{2}{\underset{l=1}{\sum }}\left(\theta_{l}\omega_{j}\right),\;\phi =\overset{2}{\underset{l=1}{\sum }}\left(\phi_{l}\omega_{j}\right).$$

Here $F_{\xi }$ is H and the shape function is defined as:

(24) $$\omega_{j}={\left(-1\right)}^{l-1}\frac{1-\frac{\xi }{\xi_{l-1}}}{1-\frac{\xi_{l}}{\xi_{l-1}}},\;l=1,2.$$

**Step II:** In this step, the matrices are stiffness, vector and boundary (integral vector). The global stiffness (matrix) is obtained whereas the Picard (linearization approach) is utilized to obtain a linear system of equations that are defined as:

(25) $$\bar{H}=\overset{2}{\underset{l=1}{\sum }}\omega \bar{H_{l}},\;\;\bar{F}=\overset{2}{\underset{l=1}{\sum }}\omega \bar{F_{l}}.$$

Here $\bar{F_{l}}$ and $\bar{H_{l}}$ are variables (nodal values).

**Step III:** The algebraic equations (non-linear) resulting from the assembly process are:

(26) $$Mat\left(F,\;H,\;\theta ,\phi \right)\left(\begin{array}{c} F\\ \theta \\ \phi \end{array}\right)=\left(F\right),$$

where (Mat) is the global stiffness matrix, (F) is the force vector and the nodal values (variables) are $\left(\begin{array}{c} F\\ \theta \\ \phi \end{array}\right).$ The Equation (18) related to the residual form is

(27) $$\left(R\right)=\left[M\left(F^{\left(r-1\right)},\;H^{\left(r-1\right)},\theta^{\left(r-1\right)},\varphi^{\left(r-1\right)}\right)\right]\left[\begin{array}{c} F^{r}\\ \theta^{r}\\ \varphi^{r} \end{array}\right]=\left[F\right],$$

(28) $$\frac{{\left(\sum_{l=1}^{N}\left(T^{r}-T^{r-1}\right)\right)}^{1/2}}{{\left(\sum_{l=1}^{N}{\left|T^{r}\right|}^{2}\right)}^{1/2}}<\frac{1}{10^{8}}.$$

**Step IV:** The computational domain is considered as [0, 8] while mesh-free analysis is computed along with 270 elements. The problem is converged at mid of each of the 270 elements. Hence, simulations of the problem are performed along with the 270 elements. Table 1 reveals the study of convergence analysis. The solution to the problem is converged after simulations of 210 to 270 elements. It is observed that 270 elements are ensured for the convergence of the problem. Outcomes are provided for velocity, concentration and temperature at mid of each of the 270 elements. All numerical simulations related to tables and graphs are captured for the 270 elements.

**Comparative analysis:** The numerical result of the current problem is verified with published results [23] by the disappearing effects of $We=\epsilon =F_{r}=H_{s}=Ec=M=D_{f}=\varphi_{1}$ and $\varphi_{2}=0.$ Numerical values of the Nusselt number are computed against the distribution in Prandtl number. Good agreements among the results of the present problem and published work [23] are presented in Table 2.

## 4. Results and Discussion

**Figure 5:**
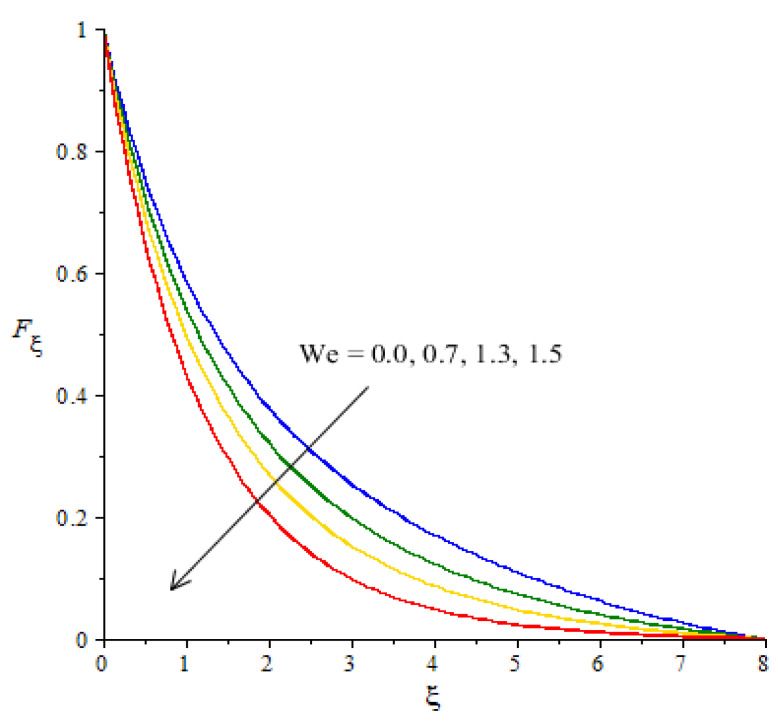
Change in velocity against We.

**Figure 6:**
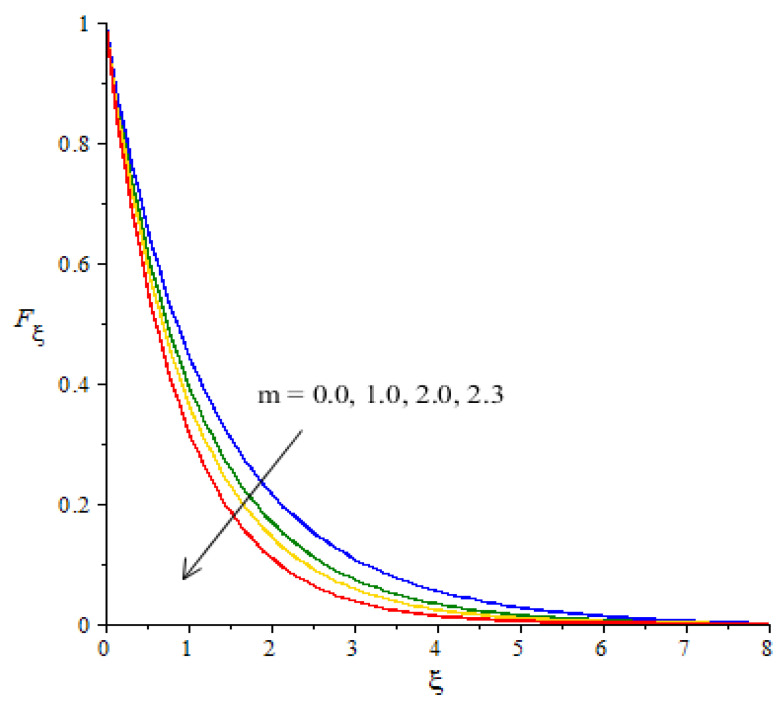
Change in velocity against m.

**Figure 7:**
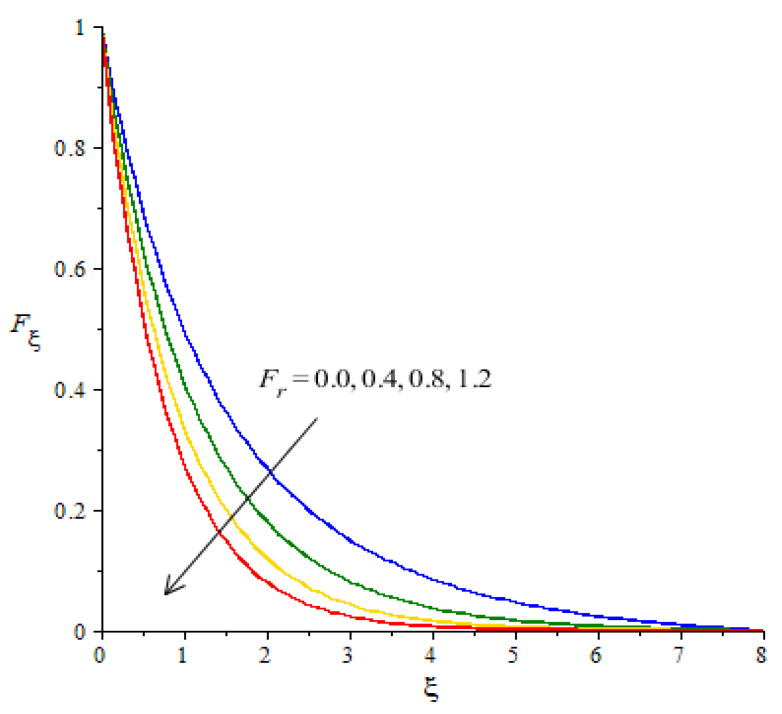
Change in velocity against $F_{r}$.

**Figure 8:**
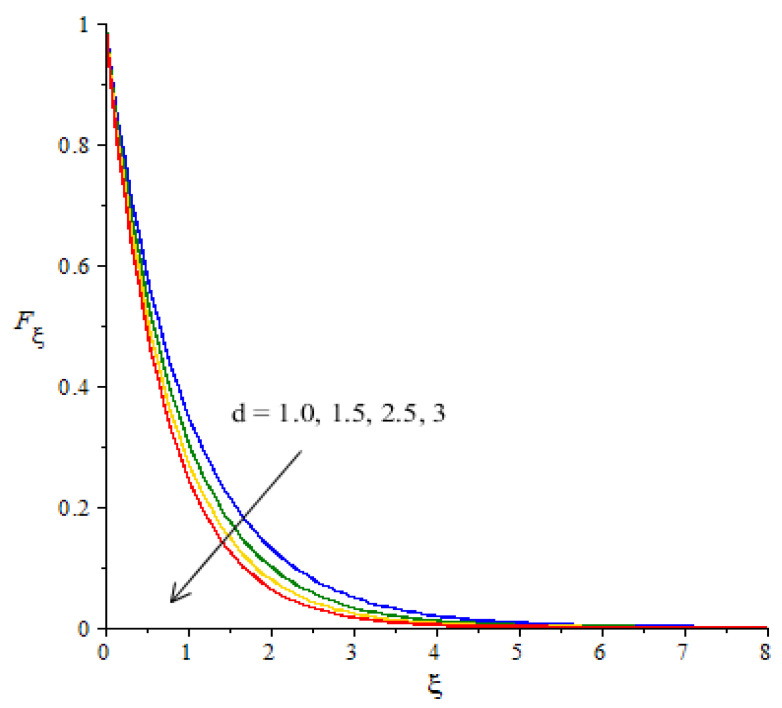
Change in velocity against d.

Mechanisms of velocity, thermal energy and diffusion of mass influenced by chemical reaction are addressed over a stretched melting surface. Correlations between silicon dioxide and Molybdenum dioxide in EG (ethylene glycol) are used in the presence of the Carreau–Yasuda liquid. Various kinds of influences (Soret, Dufour, viscous dissipation, Joule heating and magnetic field) are also addressed. As such, the complex transport phenomenon is simulated with the help of a numerical approach (FEM). The graphical computational investigations are captured in graphs and tables. The detailed outcomes are discussed below:

**Graphical investigations of velocity against distribution in various parameters:** The change in Weissenberg, power law index, Forchheimer numbers and Carreau–Yasuda variables are addressed in the motion of fluid particles considered in Figure 5, Figure 6, Figure 7 and Figure 8. Figure 5 is plotted to measure the role of We in the motion of hybrid nanoparticles. It is estimated that the motion of hybrid and fluid - nanoparticles is slowed down by applying higher We Values. The Weissenberg number is constructed in the current model due to the consideration of the rheology of the Carreau–Yasuda fluid while We is defined as a ration of elastic and viscous forces. An increase in We brings the declination in motion of fluid particles in the presence of nanoparticles and hybrid nanoparticles. Hence, a reduction is noticed versus the change in We. Moreover, the thickness of the momentum boundary layers decline when We is increased. The flow for Newtonian fluid is the dominated flow for a case of non-Newtonian fluid. The relationship between the velocity and power law index number is shown in Figure 6. The decreasing phenomenon of motion in fluid particles is captured and m is created due to tensor of the Carreau–Yasuda liquid. The numerical values of m are decided by the category of fluids (shear thinning or shear thickening). The fluid becomes thick in the case of large m values. Hence, the power law index number is not a significant parameter in the case of an enhancement in flow involvement of nanoparticles and hybrid nanoparticles. Parameter related to the power law number has a significant impact on adjusting the momentum boundary layer thickness. The role of $F_{r}$ is noticeable in the flow of nanoparticles and hybrid nanoparticles (see Figure 7). It is demonstrated that the parameter related to $F_{r}$ occurs in the momentum equation because of the Forchheimer porous. This kind of parameter behaves like a non-linear-type function in the flow of nanoparticles. In this case, the retardation force is created in fluid motion and brings resistance of the fluid particles into motion. Moreover, the thickness related to the boundary layer is reduced when large values of $F_{r}$ are applied. Further, the motion created by the Forchheimer porous is less than the motion created in the particles, excepting the involvement of Forchheimer porous media. The parameter associated with d is called the fluid variable and the change in d versus the velocity is captured in Figure 8. The large values of d create the resistance force during the flow of hybrid nanoparticles and nanoparticles. Meanwhile, the motion of fluid particles declines against the higher values of d. Momentum boundary layers have a decreasing function versus the impact of d.

**Figure 9:**
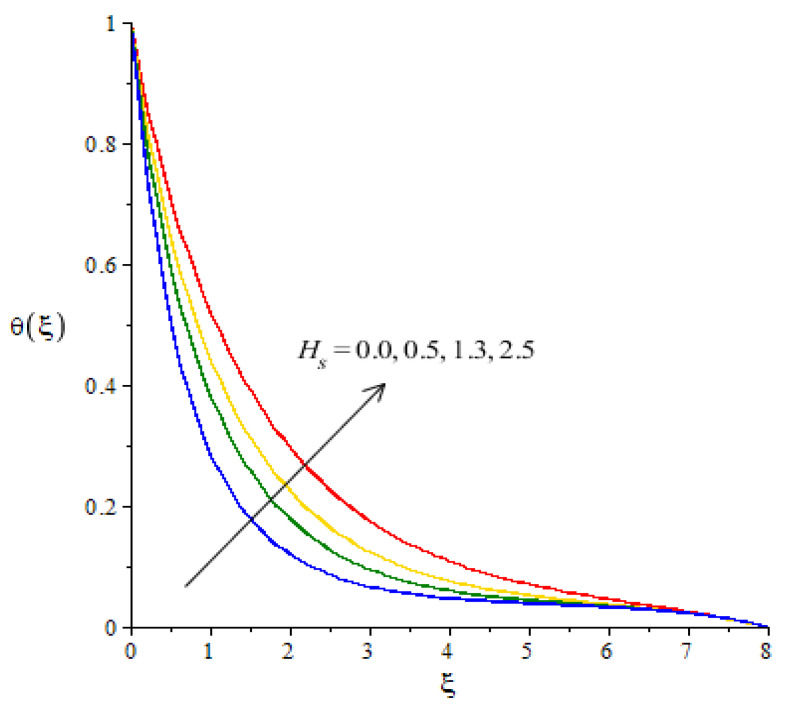
Change in thermal energy against $H_{s}$.

**Figure 10:**
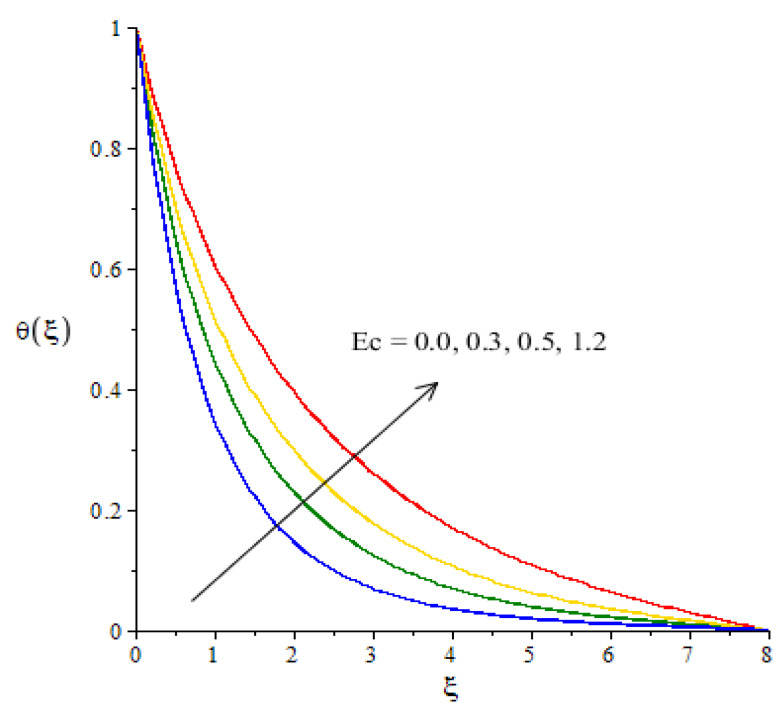
Change in thermal energy against Ec.

**Figure 11:**
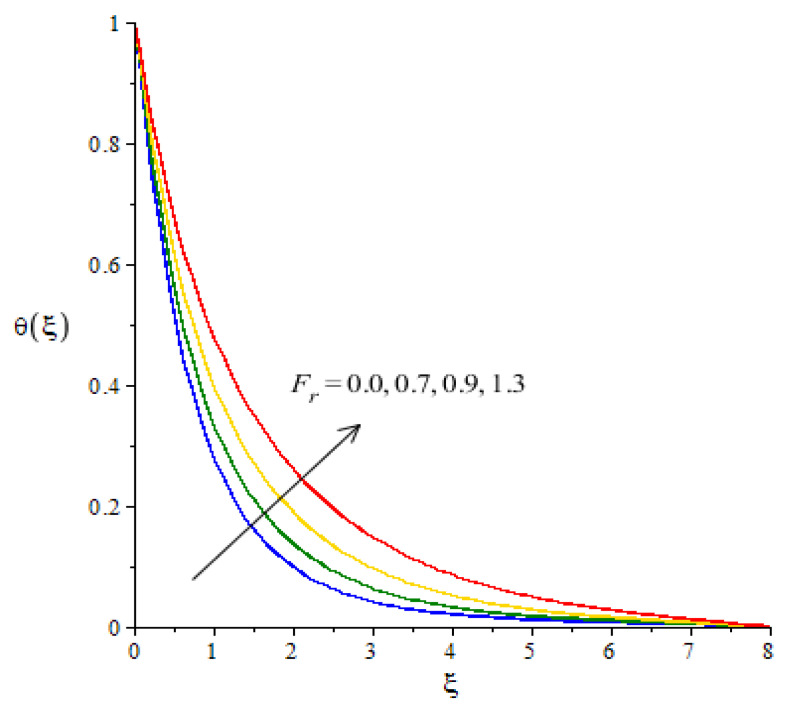
Change in thermal energy against $F_{r}$.

**Figure 12:**
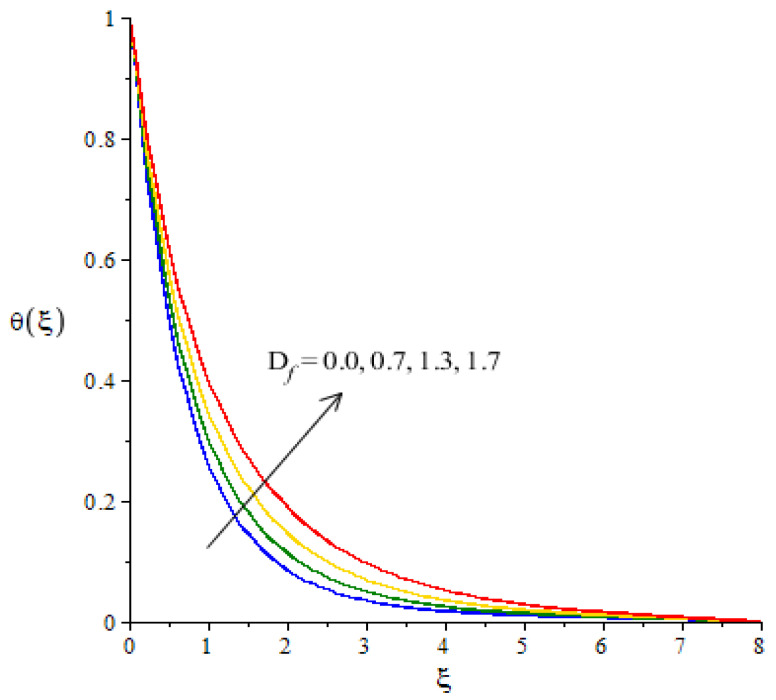
Change in thermal energy against $D_{f}$.

**Graphical investigations of heat energy against distribution in various parameters:**Figure 9, Figure 10, Figure 11 and Figure 12 reveal the characterization of thermal energy phenomenon versus the variation of $H_{s}$, $F_{r},\;Ec$ and $D_{f}.$ The thermal energy performance is measured with respect to the variation in heat generation number while this phenomena is shown in Figure 9. The production of heat energy is at its maximum when using higher values of $H_{s}$. The external heat source at the sheet surface results in the maximum production into heat energy. to Thickness related to thermal layers is also enhanced due to the large values of heat generation number. Thus, the heat generation number is a significant parameter for the maximum production of heat energy. Moreover, an inclination in thermal energy is created due to the direct relation to thermal energy. It is demonstrated that the positive values for $H_{s}$ are present due to the phenomena of heat generation. As such, the impact of heat generation is visualized in our analysis. Figure 10 plots the enhancement in heat energy against the change in Eckert number. Physically, an enhancement in heat energy due to viscous dissipation is simulated. A direct relationship between viscous dissipation and the kinetic energy phenomenon was found. The temperature of the fluid particles is enhanced due to the Eckert number. The work-done rate is enhanced by the particle heat energy when viscous dissipation occurs. The relationship between $F_{r}$ and temperature profile is visualized in Figure 11. This figure captures the better performance in heat energy, including the appearance of $F_{r}.$ In fact, $F_{r}$ is generated due to the Forchheimer porous while $F_{r}$ generates more heat energy in the presence of nanoparticles and hybrid nanoparticles. Moreover, the concept of Dufour’s number is characterized in the dimensionless heat equation due to the first law of thermodynamics while terms related to thermodynamics refer to the concept of Dufour (heat energy) because of the concentration gradient. The concentration gradient is enhanced using large values of $D_{f}.$ The fluid particles absorb more heat energy when $D_{f}$ is increased. Hence, the temperature profile is increased when there are higher values of $D_{f}$ (see Figure 12). Further, thickness of the thermal boundary layers are controlled by the impacts of $F_{r}$ and $D_{f}.$

**Figure 13:**
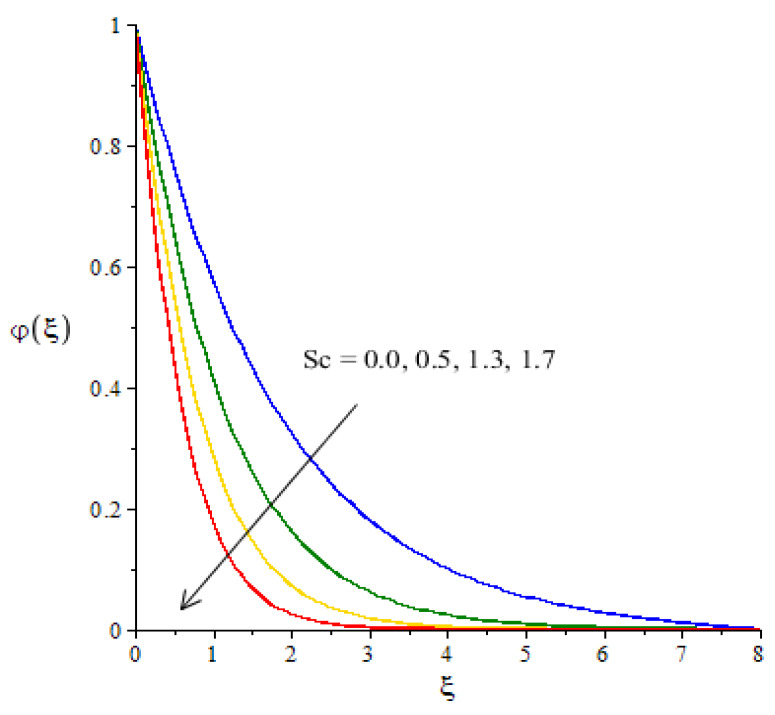
Change in concentration against Sc.

**Figure 14:**
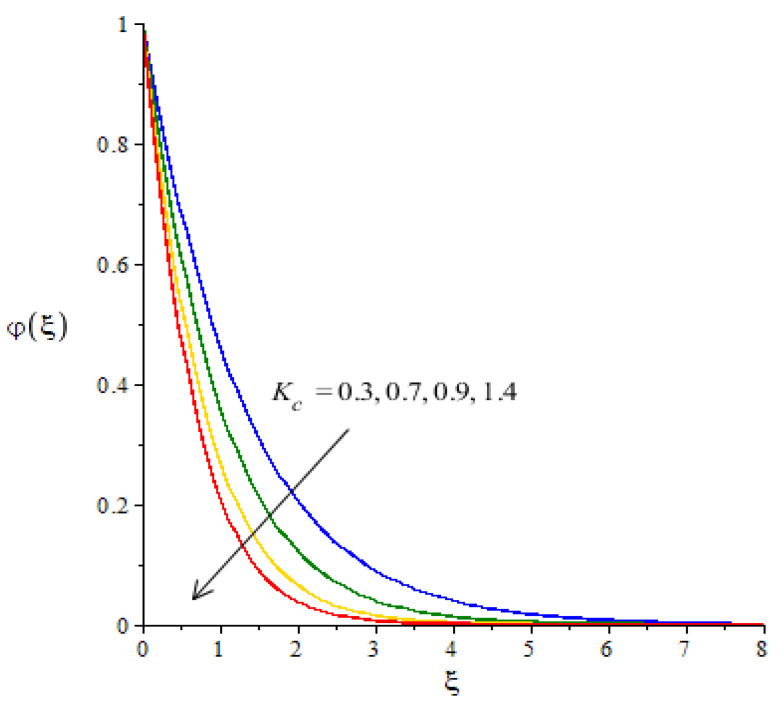
Change in concentration against $K_{c}$.

**Figure 15:**
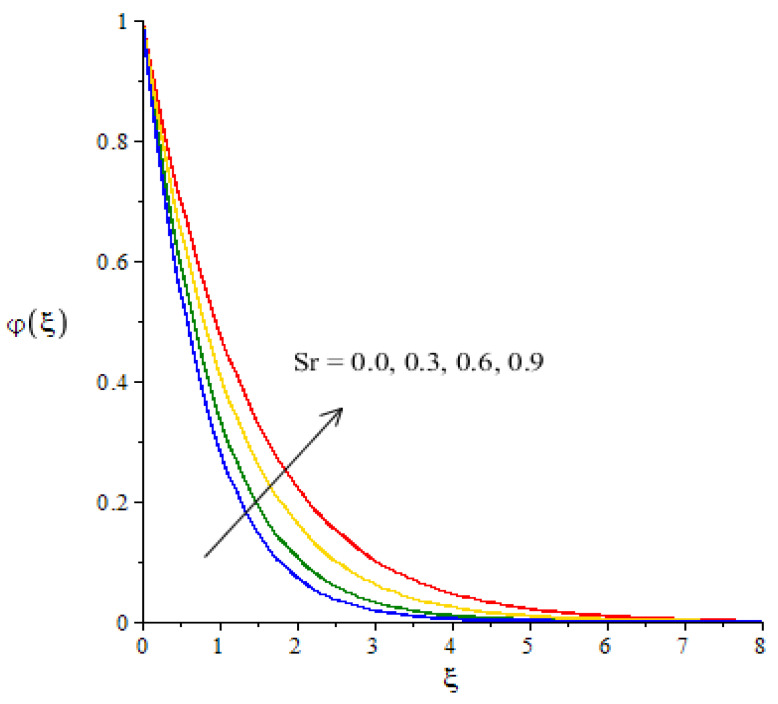
Change in concentration against Sr.

**Graphical investigations of mass diffusion against distribution in various parameters:**Figure 13, Figure 14 and Figure 15 have been plotted to visualize the transport of diffusion against the change in $Sc,\;K_{c}$ and Sr. The measurement of mass diffusion is captured in Figure 13, considering the influence of Sc. The diffusion of mass decreases when Sc is enhanced. We can observe that this reduction into mass diffusion happens due to the definition of Sc. Physically, Sc is rationed among mass and momentum diffusivities. According to the concept of Sc, the concentration curves are reduced when Sc is inclined. A similar situation occurs in terms of the boundary layer thickness in relation to concentration. Figure 14 visualizes the effect of the chemical reaction number on the transport of mass diffusion We found that the parameter related to $K_{c}$ revealed the coefficient of thermal energy along with the chemical reaction. The positive values of $K_{c}$ correspond to the destructive chemical reaction. In this case, a reduction is captured in the diffusion of the mass species. In the current flow model, the case related to a destructive chemical reaction is used. Thickness associated with the concentration layers is reduced when $K_{c}$ is increased. The decreasing graph is plotted between the Soret number and diffusion of mass as shown in Figure 15. The concept of Sr (fractioned between the difference in temperature and concentration) appears due to the temperature gradient in the concentration equation. Using Soret’s theory, the solute diffusion is enhanced due to thermal energy.

**Mechanisms of gradient temperature, surface force and mass diffusion rate versus the distribution of various parameters:** The computational analysis of surface force (skin friction coefficient), gradient temperature (Nusselt number) and rate of mass diffusion versus the variation of $D_{f}$, Sc, $H_{s}$, $F_{r}$ and We is simulated in Table 3. Table 3 reveals that the drag force (skin friction coefficient) declines when We and $F_{r}$ are increased. However, we see a reduction in the skin friction coefficient when the heat generation number is increased. When $D_{f}$ is enhanced, the constant variation is simulated in surface force at the wall. The temperature gradient decreases using the large values of $D_{f}$, $H_{s}$, $F_{r}$ and We. The parameter related to Sc plays a vital impact in maximizing the rate of thermal energy. As for the concentration gradient, it shows the same behavior as the temperature gradient.

## 5. Prime Consequences of the Problem

The transport features in the rheology of Carreau–Yasuda liquid and involvement of nanostructures and hybrid nanoparticles over a heated surface have been visualized. The Dufour and Soret effects under the action of a magnetic field have been addressed. Forchheimer porous media was also considered. The simulations of the current model were computed by finite element approach. The prime findings are captured below:

- Convergence of the problem is ensured at 270 elements;
- The motion of nanoparticles and hybrid nanoparticles in ethylene glycol is boosted versus the enhancement in fluid variable, power law index number, Weissenberg number and Forchheimer porous number;
- Significant production of heat energy versus higher values of heat generation, Eckert, Dufour and Forchheimer porous numbers;
- The transportation of solute particles declines versus the large values of Schmidt and chemical reaction numbers, but solute particles accelerate against higher values of Soret number;
- Surface force is increased via large values of Weissenberg and Forchheimer porous numbers but surface force is decreased versus the large values of heat generation number and

The role of the Schmidt number is significant in the development of temperature and concentration gradient.

## Figures and Tables

**Table 1:** Mesh-free analysis of velocity, mass diffusion and thermal energy considering 270 elements.

Number of Elements	$F_{\xi }\left(\frac{\xi_{\infty }}{2}\right)$	$\theta \left(\frac{\xi_{\infty }}{2}\right)$	$\varphi \left(\frac{\xi_{\infty }}{2}\right)$
30	0.001723308133	0.3009272684	0.5332200981
60	0.001476057680	0.2862125651	0.5165526300
90	0.001389387659	0.2814243572	0.5109967127
120	0.001345741043	0.2790514389	0.5082194520
150	0.001319507594	0.2776354377	0.5065521700
180	0.001302011520	0.2766938280	0.5054410754
210	0.001289514516	0.2760222981	0.5046476756
240	0.001280142826	0.2755193140	0.5040526977
270	0.001272856820	0.2751285026	0.5035909496

**Table 2:** Comparative simulations of Nusselt number considering by $We=\epsilon =F_{r}=H_{s}=Ec=M=D_{f}=\varphi_{1}=\varphi_{2}=0.$.

	Bilal et al. [23]	Present Results
Pr	${\left(Re\right)}^{-1/2}Nu$	${\left(Re\right)}^{-1/2}Nu$
0.07	0.0663	0.0662110383
0.20	0.1619	0.1619120330
0.70	0.4539	0.4529370132
2.00	0.9113	0.9112098201

**Table 3:** Computational analysis of surface force, gradient temperature and rate of mass diffusion versus the variation of $D_{f}$, Sc, $H_{s}$, $F_{r}$ and We in view of hybrid nanoparticles and nanoparticles.

Parameters		${\left(Re\right)}^{\frac{1}{2}}C_{f}$	${\left(Re\right)}^{-1/2}Nu$	${\left(Re\right)}^{-\frac{1}{2}}Sh$
	0.0	0.2565506571	0.9069968916	0.4191080595
We	0.4	0.3021743934	0.5709115739	0.3046713057
	0.8	0.6496811069	0.4087628063	0.1059290192
	0.3	0.3500005761	2.800587924	0.3350535080
$F_{r}$	0.5	0.5935103805	2.331989057	0.2339179505
	1.2	0.7690074135	1.272381650	0.11317865862
	0.0	0.5541216982	0.4657146871	0.99748124590
$H_{s}$	0.7	0.3541216982	0.3493805142	0.89748124590
	1.3	0.1541216982	0.1173513733	0.69748124590
	0.2	0.5541216982	0.1186981159	0.04850074987
Sc	0.4	0.5541216982	0.1186575085	0.36213534820
	0.8	0.5541216982	0.1181890841	0.57775563972
	0.3	0.5541216982	0.7182669750	0.37626972734
$D_{f}$	0.6	0.5541216982	0.5183838165	0.27404085874
	1.3	0.5541216982	0.3177686869	0.18920433118

## Data Availability

The data used to support this study are included in the Manuscript.
